# Metacognitive beliefs and their relationship with anxiety and depression in physical illnesses: A systematic review

**DOI:** 10.1371/journal.pone.0238457

**Published:** 2020-09-10

**Authors:** Lora Capobianco, Cintia Faija, Zara Husain, Adrian Wells

**Affiliations:** 1 Research and Innovation, Greater Manchester Mental Health NHS Foundation Trust, Manchester, United Kingdom; 2 Division of Nursing, Midwifery & Social Work, Faculty of Biology, Medicine and Health, Manchester Academic Health Science Centre, The University of Manchester, Manchester, United Kingdom; 3 Faculty of Biology, Medicine and Health, School of Psychological Sciences, Manchester Academic Health Science Centre, The University of Manchester, Manchester, United Kingdom; University of Vienna, AUSTRIA

## Abstract

Anxiety and depression are common among patients with chronic physical illnesses and have a significant impact on morbidity, quality of life, and health service utilisation. Psychological treatment of anxiety and depression has small to moderate efficacy in this group and is not commonly based on a model of causal mechanisms. A novel approach to understanding and improving mental health outcomes in physical illnesses is needed. One approach may be to explore the role of metacognitive beliefs which are reliably associated with anxiety and depression in individuals with mental health difficulties. The current systematic review aimed to evaluate the contribution of metacognitive beliefs to anxiety and depression across physical illnesses. Systematic searches were conducted on Web of Science, PsychINFO, MEDLINE, Embase, and CINAHL of studies published between 1997 and January 2019. 13 eligible studies were identified that in sum comprised 2851 participants. Metacognitive beliefs were found to have reliable, moderate, positive and significant associations with anxiety and depression symptoms across a range of physical illnesses. There appeared to be commonality and some specificity in the relationships. Negative metacognitive beliefs concerned with uncontrollability and danger of worry were associated with both anxiety and depression across all physical illnesses assessed, whilst more specific associations emerged for individual medical conditions where positive beliefs about worry, cognitive confidence and cognitive self-consciousness were unique correlates. Negative metacognitive beliefs of uncontrollability and danger significantly and positively predicted symptoms of anxiety and depression after controlling for factors including age, gender, disease factors and cognition (illness perceptions and intolerance of uncertainty). The results suggest that the metacognitive model of psychological disorder is applicable to psychological symptoms of anxiety and depression across a range of chronic medical conditions, implying that metacognitive therapy might be helpful in improving outcomes in multiple morbidities that involve poor mental and medical health.

## Introduction

Approximately 15 million people in England (30% of the population) have a physical illness (e.g. heart disease, diabetes) and they are two or three times more likely to experience mental health problems compared to the general population [1–10]. The presence of mental health symptoms (e.g., anxiety, depression) in physical illness has a widespread negative impact that includes poorer clinical outcomes and prognosis, an increase of adverse health behaviours (e.g. physical inactivity), poorer self-care, worse functional status and decreased quality of life [11]. In addition, those with both a mental and physical illness use more health care services [12], resulting in UK NHS spending of between 8 and 3 billion pounds [13]. Individuals with co-morbid mental health symptoms are less likely to be in employment or when they are productivity is reduced [14–16]. A better understanding of the psychological factors linked to negative mental health symptoms such as anxiety and depression in physical illness might aid in more effective psychological treatment for this population.

The National Institute for Health and Care Excellence (NICE) recommends the use of evidence-based psychological interventions to treat mental health in those with long term conditions. Among NICE recommended psychological interventions are psychoeducation, group-based skills training, individual and group cognitive behavioural therapy and pharmacological treatments [8]. Nevertheless, within-sample effect sizes for such treatments from pre to post-treatment among cardiac patients are small, ranging from Cohen’s d = 0.15 to 0.34 [17]. A recent meta-analysis among patients with a cardiovascular disease compared CBT treatment with no-intervention (n = 1), educational materials (n = 1), or usual care (n = 10). Whilst CBT significantly decreased symptoms, the effect sizes were small for depression (Cohen’s d = 0.35) and anxiety (Cohen’s d = 0.34) [18].

In cancer patients, a recent meta-analysis evaluating psychological interventions for anxiety and depression [19] found that at post-treatment psychoeducation interventions demonstrated a small within sample effect size (Cohen’s d = 0.14 for depression, and d = 0.22 for anxiety), with small to medium within sample effects for relaxation training (d = 0.37 for depression and d = 0.54 for anxiety). Psychotherapy interventions (e.g. coping skills training, CBT, supportive-expressive psychotherapy) were associated with similar post-treatment within-sample effect sizes irrespective of treatment delivery in individual or group formats (anxiety: individual psychotherapy d = 0.49, group psychotherapy d = 0.44; depression: individual psychotherapy d = 0.35, group psychotherapy d = 0.48) [19].

There are a number of reasons for the variable but often limited effects of existing treatments, including the low quality of many studies and the focus of treatments on general coping skills such as anxiety management or use of techniques that aim to reality-test negative thoughts and beliefs. Specifically, anxiety and mood disturbances are likely to be ‘normal’ following diagnosis and during invasive treatments and are part of an adjustment process. There are also barriers to the implementation of existing treatment techniques in more chronic cases of distress that might compromise effectiveness. For instance, it is not meaningful to challenge some negative beliefs and fears of recurrence in cancer and cardiac patients, many of whom are at increased risk of future health events. It is clear from treatment outcome data and the nature of existing therapies that a different approach is required. Such an approach might be grounded in modern evidence-based theories of the psychological factors that cause or maintain abnormal adjustment reactions and accompanying symptoms of anxiety and depression.

One model and treatment approach that has gained success and advanced outcomes in mental health settings is based on the Self-Regulatory Executive Function (S-REF) model [20, 21], which is the basis of metacognitive therapy [22, 23]. This approach might also offer advances in the area of physical health because unlike many exiting approaches it does not aim to teach coping skills or challenge the validity of negative thoughts about the future. Current models used in health psychology place illness perceptions in a central role in coping and the maintenance of distress. For example, Leventhal’s Common Sense Model of Self-regulation [24] suggests that there are five illness perceptions that maintain distress: identity (the illness and its symptoms), cause (beliefs about the perceived cause of the illness), time-lines (beliefs regarding how long the illness will last), consequences (beliefs about the physical and social impact of the illness on oneself), and controllability (beliefs about whether the illness can be cured or managed). Despite associations between illness perceptions and psychological outcomes [25] including reduced quality of life [25–27], the mechanisms leading to a persistence of unhelpful illness perceptions and their link with anxiety and depression remains unclear. For instance, even if an illness is perceived to be chronic, from which there can be no ‘recovery’ not everyone will develop severe or persistent anxiety or depression. What is needed is an approach that is not dependent on the content of appraisals about illness in accounting for levels of psychological distress.

The metacognitive model conceptualises emotional symptoms as part of normal recovery and focuses on modifying a specific set of psychological factors involved in the maladaptive regulation of thinking that impedes psychological adjustment. According to the model [20, 21] abnormal and persistent psychological distress results from metacognitive beliefs (i.e. beliefs about thinking) which give rise to a maladaptive thinking style termed the cognitive attentional syndrome (CAS). The CAS is characterized by negative self-referential processing such as worry, rumination, threat monitoring and coping strategies that have unintended effects. The CAS interferes with the gradual down-regulation of negative emotions and arousal following or during stressful personal experiences, such as those accompanying physical illness. The metacognitive beliefs behind the CAS are conceptualised as positive beliefs which concern the usefulness of worrying (i.e. “Worrying helps me to anticipate problems before it is too late) and negative metacognitive beliefs that focus on the uncontrollability and harmfulness of worrying (i.e. “My worrying is uncontrollable; Worrying too much will cause my cancer to return”). Negative metacognitive beliefs are considered to be of particular importance in psychological dysfunction because they lead to a sense of loss of control of thinking and a sense of current threat from cognition itself [23, 28]. One of the features of the metacognitive model is that it is transdiagnostic, suggesting that psychological distress is maintained by a common set of processes. While therapeutic interventions, namely Metacognitive Therapy (MCT), can be delivered based on disorder specific models, a generic model can also be applied. MCT focuses on regulating overthinking processes (worry and rumination) and maladaptive attention strategies using a variety of methods that includes challenging metacognitive beliefs. As such, MCT focuses on modifying the processes that maintain repetitive negative thinking rather than the content of individuals thoughts, and in doing so aids patients in becoming more flexible in dealing with their concerns.

Consistent evidence supports the hypothesised relationship between metacognitive beliefs and anxiety and depression in non-clinical and mental health populations. A recent meta-analysis found that five dimensions of metacognitions were prevalent among patients with mental health disorders [29], consistent with central predictions of the metacognitive model [20, 21]. Specifically, Sun et al [29] reported large effects of negative metacognitive beliefs concerning uncontrollability and danger, and beliefs regarding the need for control across psychiatric diagnoses (e.g. major depression, generalised anxiety disorder, obsessive compulsive disorder). In contrast, positive metacognitive beliefs showed moderate but less consistent effects but there were more specific associations with low cognitive confidence and increased cognitive self-consciousness.

Fewer studies have investigated associations between metacognitive beliefs and symptoms of anxiety and depression in physical illness. The present systematic review aimed to address this gap in the evidence-base by assessing the quality and consistency of evidence for any such relationships. If an effect can be demonstrated that is similar to that found in mental health this would have translational implications and provide support for the use of metacognitive theory and therapy to treat symptoms of anxiety and depression in patients with physical illnesses.

## Methods

The methods followed the PRISMA statement for conducting and reporting systematic reviews [30]. The study is registered with PROSPERO (ID CRD42019123581).

### Search strategy

A systematic review was conducted for articles published from 1997 to January 2019. A start date of 1997 was chosen because this was the date of the first publication of the metacognitions questionnaire (MCQ) that measures the metacognitive beliefs implicated in the S-REF model. Five electronic databases were evaluated which included: Web of Science, PsychINFO, MEDLINE, Embase, and CINAHL.

Search terms were agreed through discussion with three authors (LC, CF, AW). Search terms for the metacognition variable were created to capture a range of metacognitive belief terms and measures. The search terms for metacognition included: "metacognition questionnaire" or "meta-cognition questionnaire" or "meta cognition questionnaire" or "metacognition* questionnaire" or "meta-cognition* questionnaire" or "meta cognition* questionnaire" or "metacognitive belief*" or "meta-cognitive belief*" or "meta cognitive belief*". Search terms describing psychological distress were created to encapsulate a broad range of keywords including: "psychological distress" or "emotional distress" or "emotional disorder" or "anxiety" or "depression" or "mental illness" or "mental health" or "mental disorders" or "mood" or "stress". Search terms for physical illness were not included due to the lack of consistency on the definitions and to prevent relevant articles not being retrieved. The search strategy can be found in S1 File.

### Inclusion and exclusion criteria

Studies were eligible if they were published in a peer reviewed journal and evaluated metacognitive beliefs and anxiety or depression in a physical illness. A variety of quantitative methodological designs were included in order to be as inclusive and representative as possible, this included cross-sectional studies, longitudinal designs, and experimental designs. Case series and randomized controlled trials were also eligible if they included baseline comparisons of anxiety/depression and metacognitive beliefs. Systematic reviews, meta-analyses, and qualitative studies were excluded. In addition, book chapters, conference presentations, dissertations, theoretical articles and data sets were excluded from the review. All included studies had to report on a physical illness and this was not restricted to chronic physical illnesses. All papers had to include a validated measure of metacognitive beliefs which included the Metacognition Questionnaire- 65 (MCQ-65, [31]), the Metacognition Questionnaire- 30 (MCQ-30; [32]). The MCQ-65 is a 65-item measure of metacognitive beliefs across five subscales and demonstrates good internal consistency, Cronbach alpha’s for the five subscales are: (1) positive beliefs about worry (PB = .87), (2) negative beliefs (uncontrollability/danger, UD = .89), (3) superstition/punishment/need for control (NC = .74), (4) cognitive confidence (CC = .84), and (5) cognitive self-consciousness (CSC = .72). The MCQ-30 is a shortened version of the MCQ-65 with the same five factors and response format, with total scores ranging from 30 to 120. The MCQ-30 demonstrates good convergent validity, test-retest reliability and internal consistency [32–34]. The Cronbach’s alpha’s for the subscales are: CC = 0.93, PB = 0.92, CSC = 0.92, UD = 0.91 and NC = 0.72. Studies had to include a validated measure of psychological distress (anxiety, depression). Included studies had to be conducted in adults, studies evaluating children, adolescents, and caregivers were excluded. Studies had to be written in English, Italian, or Spanish to be included in the review because there was no translation service available for other languages.

### Data extraction

Data extraction was conducted by the first and second authors. Study characteristics extracted included the sample (e.g. physical illness, age, gender), the study design (i.e. cross-sectional, longitudinal, randomized controlled trial, case series), measures used to assess metacognitive beliefs (i.e. MCQ-65, MCQ-30), measures used for psychological distress (e.g. Hospital Anxiety and Depression Scale), and key findings (i.e. means, standard deviations, correlation coefficients).

### Quality assessment

All included studies were assessed for methodological quality and risk of bias using the NIH quality assessment tool for observational cohort and cross-sectional studies [35]. The tool has 14 items, with each item rated as yes, no, cannot decide, not applicable and not reported, and an overall rating of good, fair or poor is assigned. All studies were assessed by two independent raters (LC, CF). Any discrepancies were resolved through discussion by a third rater (ZH).

## Results

### Literature search results

**Fig 1:**
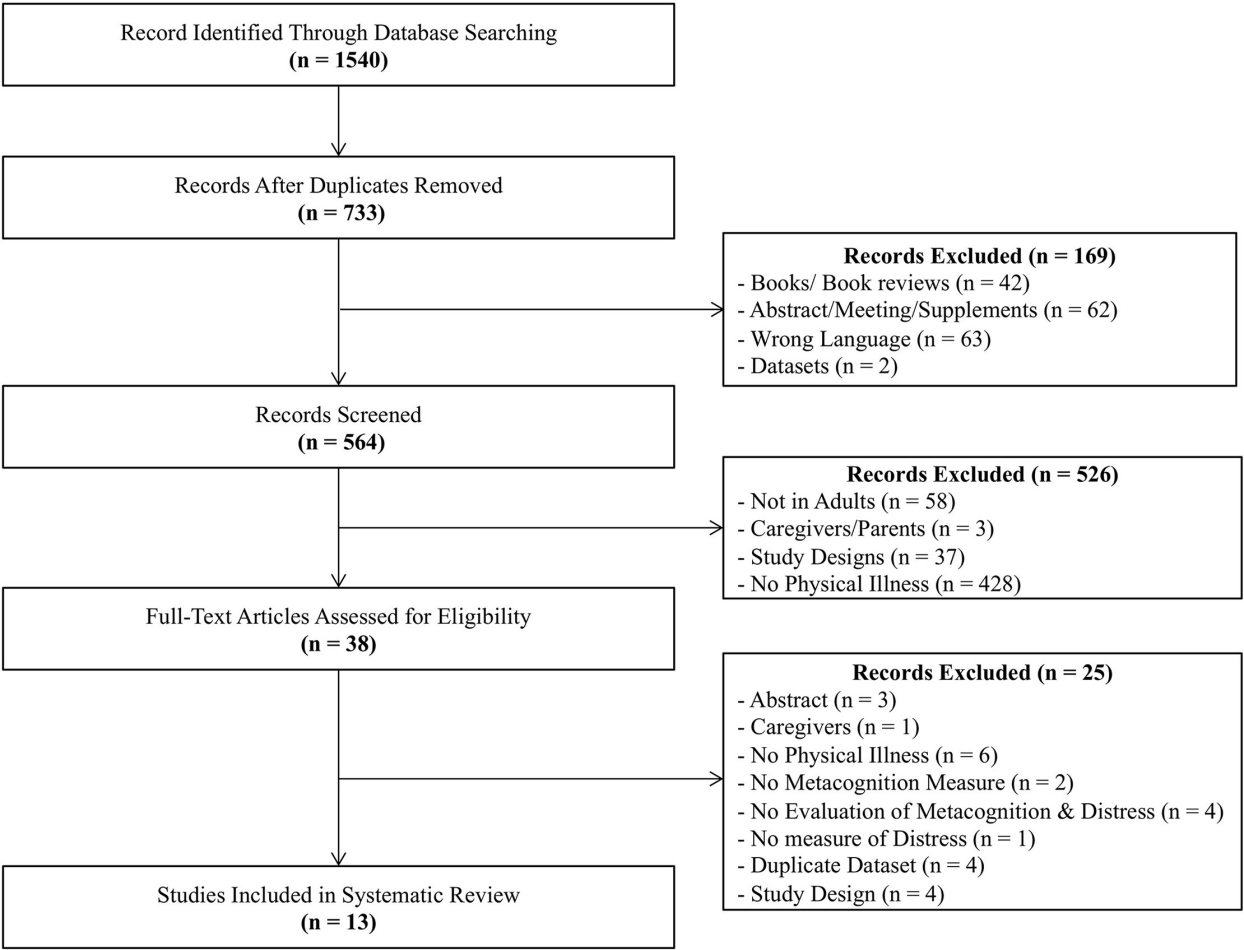
Consort diagram.

The literature search yielded 1540 papers, after removing duplicates 733 articles remained. An additional 169 articles were excluded due to being books/book reviews, conference abstracts, meetings, supplements, were in another language, or were a dataset. Following this the titles and abstracts of 564 articles were screened, which resulted in 526 articles being excluded as they were not in adults, had a study design not meeting inclusion criteria, or did not report on a physical illness. This resulted in 38 articles being assessed in full-text for eligibility. Seventeen articles were then excluded as three articles were found to be abstracts, one was on caregivers, six did not report on physical illnesses, two did not include a measure of metacognitive beliefs, four did not evaluate metacognitive beliefs and distress, and one did not include a measure of psychological distress. An additional, four were excluded [36–39] as they were case-series or randomized controlled trials that did not include baseline correlations of metacognitive beliefs and distress. A further five studies [40–44] used data from the same dataset, as such to avoid data duplication one study [41] was selected for inclusion in each case based on quality ratings and on the study aims. Fig 1 provides an overview of the screening procedure.

### Description of included studies

Of the 13 included studies, four were in cancer, two were in Parkinson’s disease, two were in epilepsy, and the remaining were in diabetes, fibromyalgia, multiple sclerosis, stroke, and cardiac samples. Overall, 2851 patients were included across studies, resulting in a mean age of 54.38 (SD = 11.23), with the overall sample being predominately female (68%). Tables 1 and 2 provide a summary of included studies, and Table 3 includes a summary of the correlations between measures of distress and metacognitive beliefs. While all studies assessed symptoms of anxiety and depression, no study included a formal mental health diagnosis. However, patients were experiencing clinically significant levels of anxiety and depression in all studies except for Donnellan et al [45] and Quattropani et al [46, 47] where patients had lower mean scores on anxiety and depression outcomes. Clinically significant anxiety and depression was defined as mean scores above cut-offs applied to symptoms scales.

**Table 1:** Description of included studies and quality assessment.

Study	Sample			Distress Measure	Key Finding	Quality Rating
	Physical Illness	Age M (SD)	Gender (M:F)			
Allot, Wells, Morrison & Walker (2005)	Parkinson’s disease	68.52 (9.61)	33:11	HADS	Metacognitive beliefs significantly and positively predicted HADS-total. NMC explained the most variance in distress.	Fair
Brown & Fernie (2015)	Parkinson’s disease	65.60 (9.30)	73:33	HADS	Three MCQ subscales were significant predictors of anxiety: NMC, PMC, CC	Fair
Compare, et al (2018)	Cardiac (Takotsubo Cardio-myopathy)	TTC-t 66.4 (12.8) TTC-nt 65.8 (11.1) AMI-t 66.1 (10.1)	12:99	HAM-D	Depression was significantly and positively associated with NMC.	Poor
Cook et al (2015)	Cancer (Breast & Prostate Cancer)	61.30 (8.90)	79:150	HADS IES	Metacognitive beliefs explained 34% of the variance in anxiety and 14% in depression. PMC and NMC were significant predictors of anxiety and depression. MCQ significant predictor of PTSD symptoms. NMC largest contributor to PTSD symptoms.	Fair
Donnellan et al (2016)	Stroke	61 (13.55)	43:21	HADS	CC, CSC, and NMC were correlated with anxiety and depression.	Fair
Fisher & Noble (2017)	Epilepsy	49 (15.40)	128:221	BDI BAI	Metacognitive beliefs explained 20% of the variance in anxiety and 24% in depression. NMC and CC significantly predicted anxiety, and NMC, CC and NC predicted depression.	Fair
Fisher et al (2018)	Cancer	20.40 (2.03)	41:46	HADS IES-R	Metacognitive beliefs were positively correlated with anxiety, depression, and PTSD. NMC showed the strongest correlation with HADS-total. NMC and CC predicted HADS-total and NMC and NC significantly predicted PSTD symptoms.	Fair
Fisher, Reilly, & Noble (2018)	Epilepsy	36.4 (12.4)	118:339	HADS	Metacognitive beliefs were associated with anxiety and depression. NMC, CC, and NTC were significant predictors of anxiety and depression	Fair
Heffer-Rahn & Fisher (2018)	Multiple Sclerosis	43.30 (11.94)	21:111	HADS	Metacognitive beliefs predicted HADS-total. NMC significant predictor of HADS-total	Fair
Kollmann, et al (2016)	Fibromyalgia	49.90 (8.50)	81:316	DASS	All the MCQ-30 subscales were positively correlated with anxiety and depression.	Poor
Purewal & Fisher (2018)	Diabetes	*T1DM* Males 50.42 (14.8) Females 43.4 2(13.9) *T2DM* Males 60.88 (10.7) Females 56.04 (12.5)	254:361	GAD-7 & PHQ-9	In T1 diabetes, NMC, CC significant predictor of anxiety and depression. NC was a significant predictor of depression. In T2 diabetes MCQ significantly predicted anxiety and depression. NMC predicted anxiety and depression. CC was also a predictor of depression.	Fair
Quattropani, Lenzo, & Filastro (2017)	Cancer (Breast Cancer)	56.09 (13.00)	0:80	HADS	NMC were highly correlated with anxiety and depression. NC also correlated with anxiety and depression. CSC correlated with anxiety not depression. NMC was the only significant predictor of anxiety, not depression	Poor
Quattropani, Lenzo, Mucciardi, & Toffle (2016)	Cancer (Breast, Colorectal, & Others)	58.21 (11.66)	34:141	HADS	NMC and CC correlated with anxiety and depression. PMC correlated with anxiety, but not depression. NMC significantly predicted anxiety and depression. CSC significantly predicted depression	Poor

HADS = Hospital Anxiety and Depression Scale; HAM-D = Hamilton Depression Scale; IES = Impact of Events Scale; BDI = Beck Depression Inventory; BAI = Beck Anxiety Inventory; DASS = Depression Anxiety Stress Scale; NMC = Negative Metacognitive Beliefs (uncontrollability and danger of worry); CC = Cognitive Confidence; CSC = Cognitive Self Consciousness; PMC = Positive Metacognitive Beliefs; NC = Need for Control; T1DM = Type 1 Diabetes Mellitus; T2DM = Type 2 Diabetes Mellitus; TTC-t = takotsubo cardiomyopathy with emotion triggers; TTC-nt = takotsubo cardiomyopathy without emotion triggers; AMI-t = acute myocardial infarction patients with emotion triggers.

**Table 2:** Means and standard deviation of distress measures and metacognitive beliefs.

Study	Physical Illness	Distress Measure M (SD)	MCQ				
			NMC M (SD)	PMC M (SD)	CSC M (SD)	CC M (SD)	NC M (SD)
Allot et al. (2005)	Parkinson’s disease	**HADS** NR	NR	NR	NR	NR	NR
Brown & Fernie (2015)	Parkinson’s disease	**HADS-A** 9.17 (2.48)	10.02 (3.75)	9.19 (3.02)	12.27 (3.33)	12.97 (4.30)	10.80 (3.20)
Compare, et al (2018)	Cardiac	**HAM-D** TTC-t 20.19 (14.4) TTC-nt 10.34 (9.3) AMI-t 18.55 (14.5)	TTC-t 21.01 (0.66) TTC-nt 13.5 (0.67) AMI-t 11.71 (0.65)	TTC-t 13.22 (0.62) TTC-nt 13.48 (0.63) AMI-t 14.00 (0.61)	TTC-t 7.52 (0.69) TTC-nt 17.35 (0.7) AMI-t 1 7.78 (0.68)	TTC-t 21.44 (0.55) TTC-nt 15.09 (0.56) AMI-t 15.03 (0.54)	TTC-t 16.87 (0.88) TTC-nt 13.54 (0.9) AMI-t 11.75 (0.88)
Cook et al (2015)	Cancer	**HADS** NR	NR	NR	NR	NR	NR
Donnellan et al (2016)	Stroke	**HADS-A** 5.66 (4.54) **HADS-D** 5.64 (4.85)	12.35 (4.87)	11.84 (5.45)	14.10 (5.18)	10.32 (4.89)	13.00 (3.76)
Fisher & Noble (2017)	Epilepsy	**BDI-II** NR **BAI** NR	NR	NR	NR	NR	NR
Fisher et al (2018)	Cancer	**HADS** 10.43 (7.55) **IES-R** 21.10 (18.15)	11.63 (5.06)	9.18 (3.65)	14.06 (4.35)	10.94 (5.13)	10.55 (3.14)
Fisher, Reilly, & Noble (2018)	Epilepsy	**HADS-A** 11.66 (4.16) **HADS-D** 7.88 (4.49)	16.19 (4.82)	10.56 (4.29)	15.94 (4.08)	16.64 (5.58)	13.35 (4.41)
Heffer-Rahn & Fisher (2018)	Multiple Sclerosis	**HADS-T** 19.46 (6.92)	14.61 (4.49)	10.84 (4.38)	16.24 (4.46)	15.08 (5.52)	11.89 (4.33)
Kollmann, et al (2016)	Fibromyalgia	**DASS-D** 16.19 (4.74) **DASS-A** 16.08 (4.9)	12.13 (4.67)	8.74 (3.28)	12.48 (3.91)	12.95 (4.94)	10.11 (3.88)
Purewal & Fisher (2018)	Diabetes	**GAD-7** 5.53 (5.44) **PHQ-9** 7.80 (6.70)	10.91 (4.83)	9.59 (3.91)	13.28 (4.58)	11.23 (5.15)	9.98 (3.81)
Quattropani et al. (2017)	Cancer	**HADS-A** 7.43 (4.34) **HADS-D** 5.86 (3.63) **HADS-T** 12.79 (7.40)	13.13 (4.63)	9.91 (4.44)	18.41 (2.89)	10.89 (4.36)	14.16 (3.44)
Quattropani et al. (2016)	Cancer	**HADS-A** 6.88 (4.32) **HADS-D** 5.76 (3.70) **HADS-T** 12.42 (7.49)	12.84 (4.53)	10.24 (4.57)	18.71 (3.38)	10.54 (4.27)	15.06 (3.47)

HADS = Hospital Anxiety and Depression Scale; BDI-II = Beck Depression Scale-II; BAI = Beck Anxiety Inventory; HAM-D = Hamilton Rating Scale for Depression; DASS = Depression, Anxiety, Stress Scale; GAD-7 = Generalized Anxiety Disorder Assessment-7; PHQ-9 = Patient Health Questionnaire -9; NMC = Negative Metacognitive Beliefs (uncontrollability and danger of worry); CC = Cognitive Confidence; CSC = Cognitive Self Consciousness; PMC = Positive Metacognitive Beliefs; NC = Need for Control; NR = Not Reported; A = Anxiety; D = Depression; T = Total; TTC-t = takotsubo cardiomyopathy with emotion triggers; TTC-nt = takotsubo cardiomyopathy without emotion triggers; AMI-t = acute myocardial infarction patients with emotion triggers.

**Table 3:** Correlation coefficients between distress measures and metacognitive beliefs.

Study	Physical Illness	Distress Measure	Correlation Coefficient Between Distress Measure & MCQ				
			NMC	PMC	CSC	CC	NC
Allot et al. (2005)	Parkinson’s disease	HADS	NR	NR	NR	NR	NR
Brown & Fernie (2015)	Parkinson’s disease	HADS-A	0.56^a^	0.38^a^	0.35^a^	0.27^a^	0.36^a^
Compare, et al (2018)	Cardiac	HAM-D	0.25^a^	-0.08	-0.09	-0.02	0.13
Cook et al (2015)	Cancer	HADS	NR	NR	NR	NR	NR
Donnellan et al (2016)	Stroke	HADS-A	0.55^a^	0.04	0.29^c^	0.42^c^	0.17
		HADS-D	0.48^a^	-0.03	0.28^c^	0.44^a^	0.18
Fisher & Noble (2017)	Epilepsy	BAI	NR	NR	NR	NR	NR
		BDI-II					
Fisher et al (2018)	Cancer	HADS	0.74^b^	0.47^b^	0.47^b^	0.46^b^	0.52^b^
		IES-R	0.70^b^	0.40^b^	0.40^b^	0.43^b^	0.59^b^
Fisher, Reilly, & Noble (2018)	Epilepsy	HADS-A	0.68^b^	0.22^b^	0.32^b^	0.27^b^	0.43^b^
		HADS-D	0.39^b^	0.08	0.09	0.35^b^	0.35^b^
Heffer-Rahn & Fisher (2018)	Multiple Sclerosis	HADS-T	0.49^b^	0.22^b^	0.1	0.45^b^	0.37^b^
Kollmann, et al (2016)	Fibromyalgia	DASS-D	0.68^b^	0.32^b^	0.53^b^	0.50^b^	0.56^b^
		DASS-A	0.72^b^	0.37^b^	0.56^b^	0.59^b^	0.58^b^
		DASS-S	0.67^b^	0.36^b^	0.53^b^	0.48^b^	0.56^b^
Purewal & Fisher (2018)	Diabetes	GAD-7	0.77^b^	0.39^b^	0.43^b^	0.43^b^	0.59^b^
		PHQ-9	0.68^b^	0.32^b^	0.39^b^	0.47^b^	0.56^b^
Quattropani et al. (2017)	Cancer	HADS-A	0.76^b^	0.19	0.30^b^	0.21	0.35^c^
		HADS-D	0.54^b^	-0.08	0.06	0.20	0.31^b^
		HADS-T	0.68^b^	0.08	0.26^c^	0.26^c^	0.38^b^
Quattropani et al. (2016)	Cancer	HADS-A	0.74^b^	0.20^c^	0.06	0.24^b^	0.09
		HADS-D	0.58^b^	0.01	-0.02	0.22^b^	0.05
		HADS-T	0.69	0.12	0.05	0.26^b^	0.10
Average Pearson’s r		Anxiety	0.68	0.31	0.36	0.37	0.46
		Depression	0.51	0.32	0.40	0.40	0.45
		Total	0.64	0.35	0.37	0.36	0.42

a = p < 0.001

b = p < 0.01

c = p < 0.05; HADS = Hospital Anxiety and Depression Scale; DASS = Depression Anxiety and Stress Scale; IES-R = Impact of Events Scale Revised; GAD-7 = Generalized Anxiety Disorder Assessment; PHQ-9 = Patient Health Questionnaire -9; BAI = Beck Anxiety Inventory; BDI-II = Beck Depression Inventory II; HAM-D = Hamilton Rating Scale for Depression; NMC = Negative Metacognitive Beliefs (uncontrollability and danger of worry); CC = Cognitive Confidence; CSC = Cognitive Self Consciousness; PMC = Positive Metacognitive Beliefs; NC = Need for Control; NR = Not Reported; A = Anxiety; D = Depression; T = Total.

All included studies used a cross-sectional design and administered the MCQ-30 as a measure of metacognitive beliefs. A range of questionnaires were used to evaluate psychological distress. The most common measure of psychological distress was the Hospital Anxiety and Depression Scale (HADS; [48]), which was used in nine studies [41, 45–47, 49–53]. One study [54] included the Depression, Anxiety, and Stress Scale (DASS; [55]), one study [56] used the Hamilton Depression Scale (HAM-D; [57]), one study [58] used the Generalized Anxiety Disorder-7 (GAD-7; [59]), and Patients Health Questionnaire-9 (PHQ-9; [60]), and one study [61] used the Beck Depression Inventory (BDI-II; [62]) and Beck Anxiety Inventory (BAI; [63]). Only one study evaluated symptoms of Post-Traumatic Stress Disorder (PTSD) using the Impact of Events Scale (IES; [64]).

Studies were assessed for methodological quality and risk of bias. Four studies were rated as poor, and nine were rated as fair. Studies rated as poor were often missing the inclusion and exclusion criteria and did not include details on the sample size calculation. Studies rated as fair often lacked descriptive details on the sample and did not report all means and standard deviations for relevant study variables. As only four studies were rated as poor, two of which were by the same author, no weighting was provided to studies based on quality ratings.

### Are metacognitive beliefs associated with anxiety and depression in physical illnesses?

#### Negative metacognitive beliefs concerning uncontrollability & danger

Negative metacognitive beliefs in the uncontrollability and danger domain were positively associated with anxiety and depression across all physical illnesses included in the review [45–47, 50–54, 56, 58] as noted in Table 3. The average correlation coefficient with anxiety was 0.68 (r = 0.55–0.77) as measured by the HADS, DASS, and GAD-7 [45–47, 50–52, 54, 58].

Similarly, these negative metacognitive beliefs were positively and significantly associated with depression when measured by the HAM-D, HADS, and PHQ-9 in cardiac, cancer, stroke, epilepsy, diabetes, and fibromyalgia patients [45–47, 52, 54, 56, 58], with an average correlation coefficient of 0.51 and a range of r = 0.25–0.68.

Negative metacognitive beliefs were also significantly and positively correlated with symptoms of PTSD r = 0.7 (measured using the IES-R), however this was only evaluated in cancer patients [41, 51].

#### Cognitive confidence

Cognitive confidence (i.e. reduced confidence in memory) was significantly and positively correlated with symptoms of anxiety and depression across each of the physical illnesses assessed except in cardiac disease and multiple sclerosis. Cognitive confidence was found to be a significant and positive correlate of anxiety, with an average Pearson’s r of 0.37 (range r = 0.24–0.59) [45, 46, 50, 52, 54, 58] as measured by the HADS, GAD-7, and the DASS. Similarly, for depression, cognitive confidence was positively and significantly correlated with depression, with an average Pearson’s r of 0.40 (range r = 0.22–0.50) [45, 46, 52, 54, 58].

#### Need for control

Need for control was significantly and positively associated with both anxiety and depression across illnesses except in stroke and cardiac patients [47, 50–54, 58]. Across studies, there was a moderate positive correlation with anxiety with an average Pearson’s r = 0.46 (range r = 0.35–0.59) and with depression with an average Pearson’s r = 0.45 (range r = 0.31–0.56).

#### Cognitive self-consciousness

Cognitive self-consciousness was positively and significantly associated with both anxiety and depression in Parkinson’s disease, stroke, cancer, epilepsy, fibromyalgia, and diabetes [45, 47, 50–52, 54, 58]. Across studies there was a positive and moderate correlation with anxiety with an average Pearson’s r = 0.36 (range = 0.29–0.56). Similarly, for depression there was a positive and moderate correlation with an average Pearson’s r = 0.40 (range = 0.28–0.53).

#### Positive metacognitive beliefs

Positive metacognitive beliefs were positively and significantly correlated with anxiety [46, 50, 52, 54, 58] in Parkinson’s disease, epilepsy, fibromyalgia, diabetes and cancer patients (average Pearson’s r = 0.31; range r = 0.2–0.39). Positive metacognitive beliefs were also associated with depression [54, 58] in fibromyalgia and diabetes patients with a positive correlation of Pearson’s r = 0.32 for both studies. This pattern of coefficients suggests that positive metacognitive beliefs show less consistency and strength of association with anxiety and depression across illnesses compared with negative metacognitive beliefs.

### Unique metacognitive predictors of anxiety, depression, and trauma symptoms

Ten of the thirteen included papers [41, 45–47, 49, 50, 52, 53, 58, 61] conducted hierarchical regressions evaluating which metacognitive beliefs subscales were independent predictors of anxiety, depression, and/or overall distress. A range of factors were controlled for including age, gender, and disease related factors (i.e. months under chemotherapy, epilepsy characteristics). Four studies [41, 50, 52, 58] also controlled for cognitions, namely illness perceptions and intolerance of uncertainty. A summary of each analysis is described in S1–S5 Tables.

When evaluating the statistical contribution of metacognitive beliefs to anxiety after controlling for additional variables including age, gender, and disease factors, metacognitive belief subscales (entered as a block) accounted for, on average, an additional 40% of the variance in anxiety. Unique contributions amongst the block of MCQ factors were made by the negative metacognitive belief subscale of uncontrollability and danger, which was the most consistent predictor of anxiety, significantly and independently predicting anxiety across cancer, diabetes, epilepsy, stroke and Parkinson’s disease patients (range β = 0.41–0.83). Quattropani et al [47] found that negative metacognitive beliefs were the strongest predictor of distress in cancer (β = 0.83). While NMC were found to be a strong predictor of distress in all three included studies on cancer [41, 46, 47], there was a large range (β = 0.44–0.83), however this may be due to the factors controlled for in the regression analyses. Cook et al [41] controlled for illness perceptions (cognition) and found a smaller contribution of negative cognitive beliefs (β = 0.44) while Quattropani et al [46, 47] controlled for fewer factors and did not include cognition, which could account for the larger contribution of metacognitive beliefs (β = 0.77 [46]; β = 0.83 [47]). In addition to uncontrollability and danger, positive metacognitive beliefs were found to significantly predict anxiety in Parkinson’s disease (β = 0.25) and cancer (β = 0.15). Whilst, cognitive confidence was found to significantly predict anxiety in Parkinson’s, stroke, epilepsy, and diabetes (range β = 0.13–0.27), however these contributions were small.

Metacognitive beliefs significantly predicted depression in diabetes, cancer, epilepsy, and stroke patients after controlling for age, gender, and disease factors. On average, metacognitive belief subscales (entered as a block) accounted for an additional 27% of the variance in depression. Amongst the block of MCQ factors unique contributions were made by negative metacognitive beliefs regarding uncontrollability and danger in diabetes, cancer, epilepsy, and stroke patients (range β = 0.23–0.71). In addition, cognitive self-consciousness and cognitive confidence were also found to significantly predict depression. Cognitive self-consciousness significantly predicted depression in stroke, epilepsy and cancer (range β = -0.24–0.32), while cognitive confidence significantly predicted depression in stroke, epilepsy and diabetes (range β = 0.17–0.26).

Only one study examined if metacognitive beliefs predicted trauma symptoms [41]. Cook et al [41] found that after controlling for age, gender and illness perceptions, metacognitive beliefs accounted for a further 17% of the variance. Specifically, negative metacognitive beliefs of uncontrollability and danger were found to be significant independent predictors (β = 0.41).

### Is cognition or metacognition a stronger predictor of anxiety and depression symptoms?

Of note, four studies controlled for cognition (intolerance of uncertainty and illness perceptions) when evaluating if metacognitive beliefs predicted symptoms of anxiety and depression [41, 50, 52, 58]. Negative metacognitive beliefs were a stronger predictor of symptoms of anxiety and depression than cognition. Brown and Fernie [50] controlled for intolerance of uncertainty (IUS). While IUS was found to be a significant predictor of symptoms of anxiety in patients with Parkinson’s disease (β= 0.31, p < 0.001), negative metacognitive beliefs were a stronger predictor of anxiety (β = 0.45, p < 0.001). Similarly, three studies evaluated the impact of illness perceptions on symptoms of anxiety in cancer, diabetes and epilepsy [41, 52, 58]. Although certain cognitions regarding illness perceptions were found to be a significant predictor of anxiety (β = -0.17–0.16, p < 0.05), metacognitive beliefs (entered as a block) were found to be a stronger predictor of anxiety symptoms. Unique contributions were made by negative metacognitive beliefs of uncontrollability and danger (β = 0.44–0.73, p < 0.001) across patients in cancer, diabetes, and epilepsy. However, Cook et al [41] found that the psychological cause subscale significantly predicted symptoms of anxiety to the same magnitude as negative metacognitive beliefs (β = 0.44).

A similar pattern emerged when evaluating symptoms of depression [41, 52, 58], whereby negative metacognitive beliefs were a stronger predictor of depressive symptoms than cognition (illness perceptions). Illness perceptions were found to significantly predict depressive symptoms in epilepsy and diabetes patients (β = 0.15–0.18, p < 0.001, [52, 58]), but not in cancer patients [41]. Unique contributions were made by negative metacognitive beliefs regarding uncontrollability and danger, which was found to be a large, positive and significant predictor of depressive symptoms (β = 0.23–0.71, p < 0.001). In addition, cognitive confidence was also found to be a significant predictor of depression (β = 0.19–0.26, p < 0.001). Whilst these results suggest that metacognitions may be more consistent and unique correlates of anxiety and depression than cognitions, they must be regarded cautiously as they are based on only four studies.

## Discussion

Metacognitive beliefs measured using the metacognitions questionnaire were found to be positively and significantly associated with anxiety, depression, and trauma across a range of physical illnesses (i.e. cancer, Parkinson’s disease, cardiac, stroke, epilepsy, multiple sclerosis, fibromyalgia and diabetes). Negative metacognitive beliefs focusing on the uncontrollability and danger of worry emerged as the subscale that was associated most consistently with both anxiety and depression. The positive relationship was observed across each of the physical illnesses assessed. Only two studies [41, 51] evaluated the association between metacognitive beliefs and trauma symptoms, finding a significant and positive correlation between trauma symptoms and negative metacognitive beliefs of uncontrollability and danger.

While the majority of correlations were moderate to large there were a few studies that reported small correlations between psychological distress measures and metacognitive beliefs. Compare et al [56], reported a significant but small correlation (r = 0.25) with uncontrollability and danger in cardiac patients. While the mean scores on the HAM-D were indicative of moderate depression, there was a large standard deviation indicating a large range in symptoms. In addition, the National Audit of Cardiac Rehabilitation [65] highlights that cardiac patients often experience greater symptoms of anxiety than depression, as such it may be that patients were predominantly anxious, however this is unclear as the study did not assess anxiety symptoms.

The relationships observed in the studies reviewed and their consistency suggests that metacognitions should be considered as potential predictors of anxiety and depression across physical illnesses. Furthermore, metacognitions concerning the uncontrollability and danger of thoughts in particular appear to be more robust correlates than cognitions, but caution is needed in this latter respect as it is based on relatively few studies. The results imply that the S-REF model of psychological disorder symptoms, that places metacognitions incentre-stage may be applicable to formulating psychological symptoms of anxiety and depression across physical illness. The robust and reliable associations across illnesses found for beliefs about uncontrollability and danger in particular are consistent with the emphasis on this factor in understanding and treating psychological disorder in metacognitive therapy [23]. The results provide further support for the transdiagnostic nature of metacognitive beliefs and their association with emotional maladaptation by extending this finding to a range of medical conditions.

A number of studies in this review controlled for gender, age disease-related factors and cognitions in testing the individual additional contribution of metacognitive beliefs to anxiety and depression [41, 45–47, 49, 50, 52, 53, 58, 61]. These results showed that the relationships found with metacognitions remained when controlling for these variables. Of particular interest when controlling for cognitive factors such as intolerance of uncertainty or illness perceptions metacognitions continued to be moderate to strong predictors of anxiety and also positive but slightly weaker predictors of depression. Specifically, metacognitive beliefs in the uncontrollability and danger domain were found to significantly predict symptoms of anxiety in patients with Parkinson’s disease, epilepsy, cancer, and diabetes. Only one study [41] found that cognition predicted anxiety symptoms to the same magnitude as metacognitive beliefs, however this scale was created by the authors and may conflate behavioural and psychological attributions. In addition, cognitive confidence was also found to significantly predict depressive symptoms in epilepsy and diabetes [52, 58]. This result is consistent with other findings where decreased cognitive confidence has been associated with depression and increased rumination (e.g. Papageorgiou & Wells [66]).

Taken together the results suggest that negative metacognitive beliefs of uncontrollability and danger may be common or universal predictors of anxiety and depression but that there are illness-specific metacognitive beliefs that may also be important. Furthermore, the relationships observed are robust against controlling for a range of factors including the influence of cognition. This is important when considering psychological interventions, suggesting that interventions targeting metacognitive beliefs may be more helpful than those that target cognition. Preliminary studies of metacognitive therapy for treating anxiety and depression within physical illnesses show promising evidence [67–69]. Fisher et al [68] evaluated metacognitive therapy for emotional distress in an open trial for adult cancer survivors. Winter et al [69] conducted a case study of MCT for adjustment disorder in a patient with pulmonary arterial hypertension. These initial studies suggest that MCT is an acceptable and feasible intervention that was associated with positive outcomes.

The results of the review mirror findings in mental health settings, which demonstrate that metacognitive beliefs are associated with anxiety and depression [29, 70–77] and that negative metacognitive beliefs of uncontrollability and danger show the strongest and most reliable relationships with these symptoms [29]. It seems therefore, that the relationships observed between anxiety, depression and metacognitions in non-clinical and mental health populations are similar to those found in patients with a range of physical health conditions. In addition, the results are in line with a systematic review conducted by Lenzo, Sardella, Martino, and Quattropani [78] in individuals with chronic medical conditions where metacognitive beliefs were associated with anxiety, depression, and quality of life. The present study extends these findings with broader inclusion and exclusion criteria (e.g., wider age range and study designs), use of a different quality assessment tool and by reporting coefficients of association between metacognitions, anxiety and depression and examining relationship when other variables were controlled.

While all metacognitive belief subscales were associated with anxiety or depression at a bivariate level it appears that a smaller set of specific metacognitions emerge as independent predictors in specific physical illnesses. This is consistent with findings in patients with varying mental health disorders (e.g. major depression, generalised anxiety disorder, obsessive compulsive disorder), where negative metacognitive beliefs concerning uncontrollability and danger were strongly associated with a range of psychiatric diagnoses. Whilst more specific associations with psychiatric disorder and low cognitive confidence and increased cognitive self-consciousness have been found [29]. This may be of particular importance for clinical applications, highlighting that across physical illnesses it appears important to consider negative metacognitive beliefs regarding uncontrollability and danger. These appear to be transdiagnostic such that irrespective of physical illness they are significant predictors of anxiety and depression. However, additional metacognitive beliefs may need to be considered based on type of illness, for example, positive beliefs about worry made additional contributions to anxiety in Parkinson’s disease and cancer. Low cognitive confidence contributed additionally to anxiety and depression in stroke, epilepsy and diabetes, whilst cognitive self-consciousness explained variance in depression in stroke, cancer and epilepsy.

While all studies assessed symptoms of anxiety and depression, no study included a formal mental health diagnosis, despite most patients experiencing clinically significant levels of anxiety and depression. Mean scores on measures of anxiety and depression indicate that the symptom severity score was above the clinical cut-off for the measure. While we do not know if patients have a diagnosed disorder, as they did not complete a diagnostic screening tool, the mean symptom scores indicate that as a group they were experiencing mild-moderate symptoms of distress. In addition, four studies [41, 52, 53, 58] reported a breakdown of the percentage of participants classed as meeting case-ness (e.g., minimum of mild anxiety/depression). Out of the 1432 participants in these four studies 27.9% met case-ness for depression, and 55.9% met case-ness for anxiety, indicating that there is a high number of patients with physical illnesses experiencing clinically significant symptoms of distress. These results suggest that the relationships observed are likely to be applicable to a range of anxiety and depression symptom severities but more research on individuals meeting clinical case-ness or diagnostic criteria for anxiety disorders and depression is needed.

While the majority of studies were rated fair quality there were studies with poor ratings, which made it difficult to assess the reliability of the associations found within these papers. For example, Compare et al [56] evaluated metacognitive beliefs in cardiac patients and noted that negative metacognitive beliefs were most strongly associated with symptoms of depression. While the association of negative metacognitive beliefs and depression is unsurprising, it is interesting that this study only evaluated symptoms of depression and not anxiety, given that cardiac patients often demonstrate higher levels of anxiety than depression [65]. Three studies did not provide descriptive data of metacognitive belief subscales or of measures of distress, which caused difficulties when interpreting study results. For example, some studies did not report means and standard deviations on measures of distress; therefore, it was unclear if participants were experiencing clinically significant levels. As such, more rigorous assessments and better descriptions of variables in studies is required. A limitation of the included studies is the restricted range of factors that were controlled for in the regression analyses predicting distress. For example, not all papers controlled for somatic factors when investigating the impact of metacognitive beliefs on anxiety and depression. As such, future studies should control for disease and related factors that may impinge on the relationship between distress and metacognitive beliefs.

One of the of limitations is that all studies were of cross-sectional design, as such information on prospective relationships between metacognition and symptoms and longer-term effects of beliefs on psychological functioning could not be evaluated. In addition, the review was limited to adults and therefore the implications of metacognitive beliefs for distress across the life-span could not be examined. However, preliminary evaluations of metacognitive beliefs in children and adolescent non-clinical samples highlight that similar to adults, metacognitive beliefs positively relate to anxiety and depression [79, 80]. In the current review we did not aim to assess the relationship between quality of life and metacognitions, but this has been assessed in an earlier review [78]. While the majority of correlations were moderate to large there were a few studies that reported small correlations between psychological distress measures and metacognitive beliefs, which may limit the findings. However, these weaker correlations were likely due to a significant proportion of the variance being accounted for by the strong relationships between negative metacognitive beliefs regarding uncontrollability and danger and psychological distress. This may suggest that negative metacognitive beliefs regarding uncontrollability and danger may be an important factor in maintaining distress across physical illnesses while additional metacognitive beliefs may be more prominent based on type of illness.

In conclusion, the results of this review show theoretically consistent positive relationships between dimensions of metacognitive beliefs and symptoms of anxiety and depression in patients with physical illnesses. The results support further exploration of these relationships coupled with more rigorous reporting of sample characteristics, descriptive statistics of the measures used, and stronger control of illness related factors. Future studies should aim to recruit clinically anxious and depressed samples of patients with physical illnesses. The field would be advanced by use of approaches to analysis that could identify the generic and specific metacognitive dimensions that contribute to anxiety and depression across different illness groups. Never the less, the results suggest that metacognitive therapy might be effectively applied in both mental and physical health settings in patients with physical and mental health co-morbidities.

## Supporting information

S1 Checklist PRISMA 2019 checklist. (DOC)

S1 File Search strategy. (DOCX)

S1 Table Metacognitive predictors of anxiety after controlling for a range of variables. (DOCX)

S2 Table Metacognitive predictors of depression after controlling for a range of variables. (DOCX)

S3 Table Metacognitive predictors of overall psychological distress. (DOCX)

S4 Table Cognitive and metacognitive predictors of anxiety. (DOCX)

S5 Table Cognitive and metacognitive predictors of depression. (DOCX)

## References

[1] Benton T, Staab J, Evans DL. Medical co-morbidity in depressive disorders. *Annals of Clinical Psychiatry*. 2007; 19(4): 289–303. 10.3109/10401230701653542 18058286

[2] Cimpean D, Drake RE. Treating co-morbid medical conditions and anxiety/depression. *Epidemiology and Psychiatric Sciences*, 2011; 20(2): 141–50. 10.1017/S2045796011000345 21714361

[3] Delahanty LM, Grant RW, Wittenberg E, Bosch JL, Wexler DJ, Cagliero E, et al Association of diabetes-related emotional distress with diabetes treatment in primary care patients with Type 2 diabetes. *Diabetic Medicine*, 2007;24 (1): 48–54. 10.1111/j.1464-5491.2007.02028.x 17227324

[4] Department of Health. Report: Long-term conditions compendium of Information: 3rd edition. 2012. Available From: https://www.gov.uk/government/publications/long-term-conditions-compendium-of-information-third-edition

[5] Fenton WS, Stover ES. Mood disorders: cardiovascular and diabetes comorbidity. *Current Opinion in Psychiatry*, 2006;19(4): 421–7. 10.1097/01.yco.0000228765.33356.9f 16721175

[6] Gunn JM, Ayton DR, Densley K, Pallant JF, Chondros P, Herrman HE, et al The association between chronic illness, multimorbidity and depressive symptoms in an Australian primary care cohort. *Social Psychiatry and Psychiatric Epidemiology*. 2010; 47(3): 175–84. 10.1007/s00127-010-0330-z 21184214

[7] Goodwin RD, Davidson KW, Keyes K. Mental disorders and cardiovascular disease among adults in the United States. *Journal of Psychiatric Research*. 2009;43(3): 239–46. 10.1016/j.jpsychires.2008.05.006 18614179 PMC3340909

[8] National Institute for Health and Care Excellence (NICE). Depression in adults with a chronic physical health problem: treatment and management. 2009 Available from: http://www.nice.org.uk/nicemedia/pdf/CG91FullGuideline.pdf 31886974

[9] Vamos EP, Mucsi I, Keszei A, Kopp MS, Novak M. Comorbid depression is associated with increased healthcare utilization and lost productivity in persons with diabetes: a large nationally representative Hungarian population survey. *Psychosomatic Medicine*. 2009;71 (5): 501–7. 10.1097/PSY.0b013e3181a5a7ad 19528291

[10] Welch CA, Czerwinski D, Ghimire B, Bertsimas D. Depression and costs of health care. *Psychosomatics*, 2009; 50 (4): 392–401. 10.1176/appi.psy.50.4.392 19687180

[11] Moussavi S, Chatterji S, Verdes E, Tandon A, Patel V, Ustun B. ‘Depression, chronic diseases, and decrements in health: results from the World Health Surveys’. *The Lancet*. 2007; 370 (9590): 851–858. 10.1016/S0140-6736(07)61415-9 17826170

[12] Dickens C, Wayne K, Blakemore A, Khara A, McGowan L, Tomenson B, et al Does depression predict the use of urgent and unscheduled care by people with long term conditions? A systematic review with meta-analysis. *Journal of psychosomatic research*. 2012; 73(5):334–342. 10.1016/j.jpsychores.2012.08.018 23062805

[13] Department of Health. Improving the Health and Well-being of People with Longterm Conditions World class services for people with long-term conditions: Information tool for commissioners. London: Department of Health 2010.

[14] Druss BG, Rosenheck RA, Sledge WH. Health and disability costs of depressive illness in a major U.S. corporation. *American Journal of Psychiatry*, 2000; 157 (8): 1274–8. 10.1176/appi.ajp.157.8.1274 10910790

[15] Hutter N, Schnurr A, Baumeister H. Healthcare costs in patients with diabetes mellitus and comorbid mental disorders–a systematic review. *Diabetologia*. 2010; 53 (12): 2470–9. 10.1007/s00125-010-1873-y 20700575

[16] Von Korff M, Katon W, Lin E.H., Simon G., Ciechanowski P., Ludman E., et al Work disability among individuals with diabetes. *Diabetes Care*. 2005;28 (6):1326–32. 10.2337/diacare.28.6.1326 15920047

[17] Dickens C, Cherrington A, Adeyemi I, Roughley K, Bower P, Garrett C, et al Characteristics of Psychological Interventions That Improve Depression in People With Coronary Heart Disease: A Systematic Review and Meta-Regression. *Psychosomatic Medicine*. 2013;75 (2):211–221. 10.1097/PSY.0b013e31827ac009 23324874

[18] Reavell J, Hopkinson M, Clarkesmith D, Lane D.A. Effectiveness of Cognitive Behavioural Therapy for Depression and Anxiety in Patients with Cardiovascular Disease A Systematic Review and Meta-Analysis. *Psychosomatic Medicine*. 2018;80 (8):742–753. 10.1097/PSY.0000000000000626 30281027

[19] Faller H, Schuler M, Richard M, Heckl U, Weis J, Kuffner R. Effects of Psycho-Oncologic Interventions on Emotional Distress and Quality of Life in Adult Patients With Cancer: Systematic Review and Meta-Analysis. *Journal of Clinical Oncology*. 2013;6:782–793. 10.1200/JCO.2011.40.8922 23319686

[20] Wells A, Matthews G. Attention and Emotion: A clinical perspective. Hove: Erlbaum 1994.

[21] Wells A, Matthews G. Modelling cognition in emotional disorder: the S-REF model. Behav. Res. Ther. 1996;34(11–12): 881–888. 10.1016/S0005-7967(96)00050-2 8990539

[22] Wells A. Emotional disorders and metacognition: Innovative cognitive therapy. Chichester, UK: Wiley 2000.

[23] Wells A. Metacognitive Therapy for Anxiety and Depression. New York, NY: Guilford press 2009.

[24] Leventhal H, Meyer D, Nerentz DR. The common sense representation of illness danger Rachman S. (Ed.) Contributions to MedicalPsychology. Oxford: Pergamon;1980 pp. 7–30.

[25] Dempster M, Howell D, McCorry NK. Illness perceptions and coping in physical health conditions: A meta-analysis. *Journal of Psychosomatic Research*. 2015; 79(6): 506–513. 10.1016/j.jpsychores.2015.10.006 26541550

[26] Foxwell R, Morley C, Frizelle. Illness perceptions, mood and quality of life: A systematic review of coronary heart disease patients. *Journal of Psychosomatic Research*. 2013; 75(3): 211–222. 10.1016/j.jpsychores.2013.05.003 23972409

[27] Spain LA, Tubridy N, Kilpatrick TJ, Adams SJ, Holmes ACN. Illness perception and health-related quality of life in multiple sclerosis. *Acta Neurol Scand*. 2007; 116: 293–299. 10.1111/j.1600-0404.2007.00895.x 17850407

[28] Wells A. Breaking the Cybernetic Code: Understanding and Treating the Human Metacognitive Control System to Enhance Mental Health. *Frontiers in Psychology*.2019;10 10.3389/fpsyg.2019.02621 PMC6920120 31920769

[29] Sun A, Zhu C, So SHW. Dysfunctional metacognition across psychopathologies: A meta-analytic review. *European Psychiatry*. 2017;45:139–153. 10.1016/j.eurpsy.2017.05.029 28763680

[30] Moher D, Liberati A, Tetzlaff J, Altman DG. Preferred reporting items for systematic reviews and meta-analyses: The PRISMA statement. *PLoS Medicine*.2009;6(7):21 10.1371/journal.pmed.1000097 PMC2707599 19621072

[31] Cartwright-Hatton S, Wells A. Beliefs about worry and intrusions: the metacognitions questionnaire and its correlate. *Journal of Anxiety Disorders*,1997;11: 279–296. 10.1016/S0887-6185(97)00011-X 9220301

[32] Wells A, Cartwright-Hatton S. A short form of the metacognitions questionnaire: properties of the MCQ-30. *Behaviour Research and Therapy*.2004;42:385–396. 10.1016/S0005-7967(03)00147-5 14998733

[33] Spada MM, Mohiyeddini C, & Wells A. Measuring metacognitions associated with emotional distress: Factor structure and predictive validity of the Metacognitions Questionnaire 30. *Personality and Individual Differences*. 2008;45(3):238–242. 10.1016/j.paid.2008.04.005

[34] Yilmaz AE, Gencoz T, Wells A. Psychometric characteristics of the Penn State Worry Questionnaire and Metacognitions Questionnaire-30 and metacognitive predictors of worry and obsessive-compulsive symptoms in a Turkish sample. *Clin Psychol Psychother*. 2008; 15(6):424–39. 10.1002/cpp.589 19115461

[35] Heart National, Lung, and Blood Institute of the National Institutes of Health (NIH). Quality assessment tool for observational cohort and cross-sectional studies. 2014 Available from: http://www.NIH.gov

[36] Fisher PL, McNicol K, Young B, Smith E, Salmon P. Alleviating emotional distress in adolescent and young adult cancer survivors: An open trial of metacognitive therapy. *Journal Of Adolescent And Young Adult Oncology*. 2015; 4(2): 64–69. 10.1089/jayao.2014.0046 26812553

[37] McNicol K, Salmon P, Young B, & Fisher P. Alleviating emotional distress in a young adult survivor of adolescent cancer: A case study illustrating a new application of metacognitive therapy. *Clinical Case Studies*.2013;12(1):22–38. 10.1177/1534650112461298

[38] Fernie BA, Murphy G, Wells A, Nikčević AV, & Spada MM. Treatment Outcome and Metacognitive Change in CBT and GET for Chronic Fatigue Syndrome. *Behav Cogn Psychother*. 2016;44(4):397–409. 10.1017/S135246581500017X 25895437

[39] Lawson RA, Millar D, Brown RG, Burn DJ. Guided Self-Help for the Management of Worry in Parkinson’s Disease: A Pilot Study. *Journal of Parkinson’s Disease*. 2013;3(1):61–68. 10.3233/JPD-120156 23938312

[40] Cook SA, Salmon P, Dunn G, & Fisher P. Measuring metacognition in cancer: Validation of the Metacognitions Questionnaire 30 (MCQ-30). *PLoS ONE*. 2014;9(9): e107302 10.1371/journal.pone.0107302 25215527 PMC4162595

[41] Cook S, Salmon P, Dunn G, Holcombe C, Cornford P, Fisher P. The Association of Metacognitive Beliefs With Emotional Distress After Diagnosis of Cancer. *Health Psychology*.2015a;34(3):20–215. 10.1037/hea0000096 25133826 PMC4321533

[42] Cook SA, Salmon P, Dunn G, Holcombe C, Cornford P, & Fisher P. A Prospective Study of the Association of Metacognitive Beliefs and Processes with Persistent Emotional Distress After Diagnosis of Cancer. *Cognitive Therapy And Research*.2015b;39:51–60. 10.1007/s10608-014-9640-x 25657483 PMC4312385

[43] Fisher PL, Byrne A, & Salmon P. Metacognitive therapy for emotional distress in adult cancer survivors: A case series. *Cognitive Therapy And Research*. 2017;41(6):891–901. 10.1007/s10608-017-9862-9 29104332 PMC5656708

[44] Fisher PL, Cook SA, & Noble A. Clinical utility of the Metacognitions Questionnaire 30 in people with epilepsy. *Epilepsy & Behavior*. 2015;57:185–191. 10.1016/j.yebeh.2016.02.004 26970994

[45] Donnellan C, Al Banna M, Redha N, Al Sharoqi I, Al-Jishi A, Bakhiet M, et al Association Between metacognition and Mood Symptoms Poststroke. *Journal of Geriatric Psychiatry and Neurology*.2016;29(4): 212–220. 10.1177/0891988716640374 27056067

[46] Quattropani MC, Lenzo V, Mucciardi M, Toffle ME. Metacognition as predictor of emotional distress in cancer patients. *Life Span and Disability*.2016;2: 221–239.

[47] Quattropani M.C., Lenzo V., Filastro A. Predictive factors of anxiety and depression symptoms in patients with breast cancer undergoing chemotherapy. An explorative study based on metacognitions. *Journal of Psychopathology*. 2017;23:67–73.

[48] Zigmond AS, Snaith AP. The Hospital Anxiety and Depression Scale. *Acta Psychiatrica Scandinavica*.1983;67(6):361–370. 10.1111/j.1600-0447.1983.tb09716 6880820

[49] Allott R, Wells A, Morrison AP, Waller R. Distress in Parkinson’s disease: contributions of disease factors and metacognitive style. *BR J Psychiatry*. 2005;187(2):182–183. 10.1192/bjp.187.2.182 16055832

[50] Brown RG, Fernie BA. Metacognitions, anxiety, and distress related to motor fluctuations in Parkinson’s disease. *Journal of Psychosomatic Research*. 2015;78(2):143–148. 10.1016/j.jpsychores.2014.09.021 25311871

[51] Fisher PL, McNicol K, Cherry MG, Young B, Smith E, Abbey G, et al The association of metacognitive beliefs with emotional distress and trauma symptoms in adolescent and young adult survivors of cancer. *Journal of Psychosocial Oncology*. 2018;36(5):545–556. 10.1080/07347332.2018.1440276 29611779

[52] Fisher PL, Reilly J, Noble A. Metacognitive beliefs and illness perceptions are associated with emotional distress in people with epilepsy. *Epilepsy & Behaviour*.2018; 86:9–14. 10.1016/j.yebeh.2018.07.008 30036766

[53] Heffer-Rahn P, Fisher P.L. The clinical utility of metacognitive beliefs and processes in emotional distress in people with multiple sclerosis. *Journal of Psychosomatic Research*. 2018;104:88–94. 10.1016/j.jpsychores.2017.11.014 29275790

[54] Kollmann J, Gollwitzer M, Spada MM, Fernie BA. The association between metacognition and the impact of Fibromyalgia in a German sample. *Journal of Psychosomatic Research*. 2016;83:1–9. 10.1016/j.jpsychores.2016.02.002 27020069

[55] Lovibond PF, Lovibond SH. The structure of negative emotional states: comparison of the Depression Anxiety Stress Scales (DASS) with the Beck Depression and Anxiety Inventories. *Behav Res Ther*. 1995;33 (3):335–343. 7726811 10.1016/0005-7967(94)00075-u

[56] Compare A, Brugnera A, Spada MM, Zarbo C, Tasca GA, Sassaroli S, et al The Role of Emotional Competence in Takotsubo Cardiomyopathy. *Psychosomatic Medicine*. 2018;80:377–384. 10.1097/PSY.0000000000000564 29406323

[57] Hamilton M. Rating depressive patients. *J Clin Psychiatry*.1980;41:21–4. 7440521

[58] Purewal R, Fisher PL. The contribution of illness perceptions and metacognitive beliefs to anxiety and depression in adults with diabetes. *Diabetes Research and Clinical Practice*. 2017;136:16–22. 10.1016/j.diabres.2017.11.029 29203257

[59] Spitzer RL, Kroenke K, Williams JBW, Lowe B. A brief measure for assessing generalized anxiety disorder. *Arch Inern Med*. 2006;166:1092–1097. 10.1001/archinte.166.10.1092 16717171

[60] Kroenke K, Spitzer RL, Williams JB. The PHQ-9: validity of a brief depression severity measure. *J Gen Intern Med* 2001;16:606–13. 10.1046/j.1525-1497.2001.016009606.x 11556941 PMC1495268

[61] Fisher PL, Noble AJ. Anxiety and depression in people with epilepsy: The contribution of metacognitive beliefs. *Seizure*. 2017;50:153–159. 10.1016/j.seizure.2017.06.012 28667910

[62] Beck AT, Steer RA, Brown GK. Manual for the Beck Depression Inventory-II. San Antonio, Texas: Psychological Corporation;1996.

[63] Beck AT, Epstein N, Brown G, & Steer RA. An inventory for measuring clinical anxiety: Psychometric properties. *Journal of Consulting and Clinical Psychology*. 1988;56:893–897. 3204199 10.1037//0022-006x.56.6.893

[64] Weiss DS. & Marmar CR. The impact of event scale-revised In: Wilson JP, & Kean TM, editors. Assessing psychological trauma and PTSD: a practitioner’s handbook. New York: Guildford; 1995.ch. 15.

[65] British Heart Foundation. National Audit of Cardiac Rehabilitation (NACR). Annual Report. 2019. Available From: https://www.bhf.org.uk/informationsupport/publications/statistics/national-audit-of-cardiac-rehabilitation-quality-and-outcomes-report-2019

[66] Papageorgiou C, Wells A. An Empirical Test of a Clinical Metacognitive Model of Rumination and Depression. *Cognitive Therapy and Research*. 2003;27 (3): 261–273. 10.1023/A:1023962332399

[67] Fisher PL, Byrne A, Salmon P. Metacognitive Therapy for Emotional Distress in Adult Cancer Survivors: A Case Series. *Cognitive Therapy and Research*. 2017; 41: 891–901. 10.1007/s10608-017-9862-9 29104332 PMC5656708

[68] Fisher PL, Byrne A, Fairburn L, Ulmer H, Abbey G, Salmon P. Brief Metacognitive Therapy for Emotional Distress in Adult Cancer Survivors. *Frontiers in Psychology*, 2019;10:162 10.3389/fpsyg.2019.00162 30766505 PMC6365419

[69] Winter L, Naumann F, Olsson K, Fuge J, Hoeper MM, Kahl KG. Metacognitive Therapy for Adjustment Disorder in a Patient With Newl Diagnosed Pulmonary Arterial Hypertension: A Case Report. *Front*. *Psychol*, 2020;11:143 10.3389/fpsyg.2020.00143 32116944 PMC7028769

[70] Bennett H. & Wells A. Metacognition, memory disorganization and rumination in posttraumatic stress symptoms. *Journal of Anxiety Disorders*. 2010;24: 318–325. 10.3389/fpsyg.2020.00143 20144524

[71] Fergus TA, & Bardeen JR. Examining the Incremental Contribution of Metacognitive Beliefs Beyond Content-Specific Beliefs in Relation to Posttraumatic Stress in a Community Sample. *Psychological Trauma*: *Theory*, *Research*, *Practice*, *and Policy*. 2016;11:143 10.1037/tra0000247 27991814

[72] Halvorsen M, Hagen R, Hjemdal O, Eriksen MS, Sørli ÅJ, Waterloo K, et al Metacognition and Thought Control Strategies in Unipolar Major Depression: A Comparison of Currently Depressed, Previously Depressed, and Never- Depressed Individuals. Cognitive Therapy and Research. 2015;39(1):31–40.

[73] Hjemdal O, Stiles T, & Wells A. Automatic thoughts and meta-cognition as predictors of depressive or anxious symptoms: a prospective study of two trajectories. Scandinavian Journal of Psychology. 2013;54(2):59–65. 10.1111/sjop.12010 23253125

[74] O’Carroll P. J, & Fisher P. Metacognitions, worry and attentional control in predicting OSCE performance test anxiety. Medical Education. 2013;47:562–568. 10.1111/medu.12125 23662873

[75] Ryum T, Kennair LEO, Hjemdal O, Hagen R, Halvorsen JO, Solem S. Worry and Metacognition as Predictors of Anxiety Symptoms: A Prospective Study. Frontiers in Psychology. 2017;8:924 10.3389/fpsyg.2017.00924 28620338 PMC5450809

[76] Takarangi MKT, Smith RA, Strange D, Flowe HD. Metacognitive and Metamemory Beliefs in the Development and Maintenance of Posttraumatic Stress Disorder. Clinical Psychological Science. 2017;5(1):131–140. 10.1177/2167702616649348

[77] Yılmaz AE, Gencöz T, & Wells A. Unique contributions of metacognition and cognition to depressive symptoms. J Gen Psychol. 2015;142(1):23–33. 10.1080/00221309.2014.964658 25539184

[78] Lenzo V, Sardella A, Martino G, Quattropani MC. A Systematic Review of Metacognitve Beliefs in Chronic Medical Condition. *Frontiers in Psychol*, 2020; 10:2875 10.3389/fpsyg.2019.02875 PMC6965316 31998178

[79] Myers D.G., Wells A. Early Trauma, negative affect, and anxious attachment: the role of metacognition. *Anxiety*, *Stress & Coping*. 2015;28(6), 634–649. 10.1080/10615806.2015.1009832 25626392

[80] Reinholdt-Dunne ML, Blicher A, Nordahl H, Normann N, Esbjørn BH, & Wells A. Modelling the Relationships Between Metacognitive Beliefs, Attention Control and Symptoms in Children With and Without Anxiety Disorders: A Test of the S-REF Model. *Frontiers in psychology*. 2019;10:1205 10.3389/fpsyg.2019.012 31231273 PMC6568246

