# Supplementary material for: Metacognitive beliefs and their relationship with anxiety and depression in physical illnesses: A systematic review
Source: PLoS One. 2020 Sep 10;15(9):e0238457. doi: 10.1371/journal.pone.0238457 (PMC7500039; doi:10.1371/journal.pone.0238457)
Supplement: S1 File — (DOCX) [file pone.0238457.s002.docx]

**S1. Search Strategy**

Database: APA PsycInfo <1806 to May Week 2 2020>

Search Strategy:

--------------------------------------------------------------------------------

1 ("metacognition questionnaire" or "meta-cognition questionnaire" or "meta cognition questionnaire" or "metacognition* questionnaire" or "meta-cognition* questionnaire" or "meta cognition* questionnaire" or "metacognitive belief*" or "meta-cognitive belief*" or "meta cognitive belief*" or "MCQ-30" or "MCQ 30" or "MCQ-65" or "MCQ 65").mp. [mp=title, abstract, heading word, table of contents, key concepts, original title, tests & measures, mesh] (847)

2 limit 1 to yr="1997 -Current" (837)

3 ("psychological distress" or "emotional distress" or "emotional disorder" or "anxiety" or "depression" or "mental illness" or "mental health" or "mental disorder*" or "mood" or "stress" or "depressive").mp. [mp=title, abstract, heading word, table of contents, key concepts, original title, tests & measures, mesh] (922692)

4 limit 3 to yr="1997 -Current" (679204)

5 2 and 4 (603)
