# Supplementary material for: Metacognitive beliefs and their relationship with anxiety and depression in physical illnesses: A systematic review
Source: PLoS One. 2020 Sep 10;15(9):e0238457. doi: 10.1371/journal.pone.0238457 (PMC7500039; doi:10.1371/journal.pone.0238457)
Supplement: S1 Table — (DOCX) [file pone.0238457.s003.docx]

**S1. Metacognitive Predictors of Anxiety After Controlling for a range of Variables**

| Study | Physical Illness | Distress Measure | Factors Controlled for | ΔR^2^ | MCQ Predictor of Distress | | | | |
| --- | --- | --- | --- | --- | --- | --- | --- | --- | --- |
|  |  |  |  |  | NMC (β) | PMC (β) | CSC (β) | CC (β) | NC (β) |
| Brown & Fernie (2015) | Parkinson | **HADS-A** | - Motor Fluctuations  - Intolerance of Uncertainty | 0.16 | 0.45** | 0.25** |  | 0.27** |  |
| Cook et al (2015) | Cancer | **HADS-A** | - Age - Gender -Illness Perceptions | 0.23 | 0.44** | 0.15** |  |  |  |
| Donnellan et al (2016) | Stroke | **HADS-A** | - Education - Cognitive Impairment | 0.41 | 0.52** |  |  | 0.27* |  |
| Fisher & Noble (2017) | Epilepsy | **BAI** | - Age - Gender - Employment - Education - Epilepsy Characteristics  - Comorbidity & Medication | 0.20 | 0.41** |  |  | 0.13** |  |
| Fisher, Reilly, & Noble (2018) | Epilepsy | **HADS-A** | - Age  - Gender  - Employment  -Education  - Relationship Status  - Epilepsy Characteristics  -Illness perceptions | 0.55 | 0.54** |  |  |  |  |
| Purewal & Fisher (2018) | Diabetes | **GAD-7** | - Age  -Gender  -Illness Perceptions | Type 1  0.37  Type 2  0.33 | Type 1  0.64**  Type 2  0.73* |  |  | Type 1  0.13** |  |
| Quattropani et al. (2017) | Cancer | **HADS-A** | - Age  - Months under chemotherapy | 0.65 | 0.83** |  |  |  |  |
| Quattropani et al. (2016) | Cancer | **HADS-A** | - Age - Gender  - Months under chemotherapy | 0.53 | 0.77** |  |  |  |  |

**Note:** HADS-A= Hospital Anxiety and Depression Scale Anxiety Subscale; BAI = Beck Anxiety Inventory; GAD-7 = Generalized Anxiety Disorder Assessment; NMC = Negative Metacognitive Beliefs (uncontrollability and danger of worry); CC = Cognitive Confidence; CSC = Cognitive Self Consciousness; PMC = Positive Metacognitive Beliefs; NC = Need for Control; ** = p < 0.001; * = p < 0.05
