# Supplementary material for: Metacognitive beliefs and their relationship with anxiety and depression in physical illnesses: A systematic review
Source: PLoS One. 2020 Sep 10;15(9):e0238457. doi: 10.1371/journal.pone.0238457 (PMC7500039; doi:10.1371/journal.pone.0238457)
Supplement: S2 Table — (DOCX) [file pone.0238457.s004.docx]

**S2. Metacognitive Predictors of Depression After Controlling for a range of Variables**

| Study | Physical Illness | Distress Measure | Factors Controlled for | ΔR^2^ | MCQ Predictor of Distress | | | | |
| --- | --- | --- | --- | --- | --- | --- | --- | --- | --- |
|  |  |  |  |  | NMC (β) | PMC (β) | CSC (β) | CC (β) | NC (β) |
| Cook et al (2015) | Cancer | HADS-D | - Age  - Gender  -Illness Perceptions | 0.09 | 0.29** |  |  |  |  |
| Donnellan et al (2016) | Stroke | HADS-D | - Education  -Cognitive Impairment | 0.3 | 0.34* |  | 0.32* | 0.21* |  |
| Fisher & Noble (2017) | Epilepsy | BDI-II | - Age  - Gender  - Employment  - Education  - Epilepsy characteristic  - Comorbidity  - Medication | 0.24 | 0.39** |  |  | 0.17** | 0.14* |
| Fisher, Reilly, & Noble (2018) | Epilepsy | HADS-D | - Age  - Gender  - Employment  - Education  - Relationship Status  - Epilepsy Characteristics  - Illness Perceptions | 0.36 | 0.23 |  | -0.12* | 0.19** | 0.23** |
| Purewal & Fisher (2018) | Diabetes | PHQ-9 | - Age  - Gender  - Illness Perceptions | Type 1  0.28  Type 2  0.27 | Type 1  0.53**  Type 2  0.71 |  |  | Type 1  0.23**  Type 2  0.26** | Type 1  0.23* |
| Quattropani et al. (2017) | Cancer | HADS-D | - Age  - Months Under chemotherapy | 0.25 | 0.51* |  |  |  |  |
| Quattropani et al. (2016) | Cancer | HADS-D | - Age  - Gender  - Months under chemotherapy | 0.36 | 0.60** |  | -0.24** |  |  |

**Note:** HADS-D = Hospital Anxiety and Depression Scale Depression Subscale; BDI-II = Beck Depression Inventory II; PHQ-9 = Patient Health Questionnaire-9; NMC = Negative Metacognitive Beliefs (uncontrollability and danger of worry); CC = Cognitive Confidence; CSC = Cognitive Self Consciousness; PMC = Positive Metacognitive Beliefs; NC = Need for Control; ** = p < 0.001; * = p < 0.05
