# Supplementary material for: Metacognitive beliefs and their relationship with anxiety and depression in physical illnesses: A systematic review
Source: PLoS One. 2020 Sep 10;15(9):e0238457. doi: 10.1371/journal.pone.0238457 (PMC7500039; doi:10.1371/journal.pone.0238457)
Supplement: S3 Table — (DOCX) [file pone.0238457.s005.docx]

**S3. Metacognitive Predictors of Overall Psychological Distress**

| Study | Physical Illness | Distress Measure | Factors Controlled for | ΔR^2^ | MCQ Predictor of Distress | | | | |  |
| --- | --- | --- | --- | --- | --- | --- | --- | --- | --- | --- |
|  |  |  |  |  | NMC (β) | PMC (β) | CSC (β) | CC (β) | NC (β) | Total (β) |
| Allot et al. (2005) | Parkinson’s disease | **HADS-T** | - Disease factors | 0.37 | 0.12** |  |  |  |  |  |
| Heffer-Rahn & Fisher (2018) | Multiple Sclerosis | **HADS-T** | - Gender  - Education  -Employment  -Pain  -Fatigue  -CAS  - Timeline -Consequences  -Personal control -Treatment control -Illness coherence  -Timeline cyclical | 0.045 | 0.19* |  |  |  |  |  |
| Quattropani et al. (2017) | Cancer | **HADS-T** | - Age  - Months under chemotherapy | -0.13 | 0.72** |  |  |  |  |  |
| Quattropani et al. (2016) | Cancer | **HADS-T** | - Age  - Gender  - Months under chemotherapy | 0.49 | 0.72** |  |  |  |  |  |

**Note:** HADS-T = Hospital Anxiety and Depression Scale Total Score; NMC = Negative Metacognitive Beliefs (uncontrollability and danger of worry); CAS = Cognitive Attentional Syndrome; CC = Cognitive Confidence; CSC = Cognitive Self Consciousness; PMC = Positive Metacognitive Beliefs; NC = Need for Control; ** = p < 0.001; * = p < 0.05
