# Supplementary material for: Metacognitive beliefs and their relationship with anxiety and depression in physical illnesses: A systematic review
Source: PLoS One. 2020 Sep 10;15(9):e0238457. doi: 10.1371/journal.pone.0238457 (PMC7500039; doi:10.1371/journal.pone.0238457)
Supplement: S4 Table — (DOCX) [file pone.0238457.s006.docx]

**S4.** Cognitive and Metacognitive Predictors of Anxiety

|  | Brown & Fernie (2015) | Cook et al (2015) | Fisher, Reilly & Noble (2018) | Purewal & Fisher (2018) | |
| --- | --- | --- | --- | --- | --- |
|  | Parkinson’s Disease  (β) | Cancer (β) | Epilepsy (β) | Type 1 Diabetes (β) | Type 2 Diabetes (β) |
| Intolerance of Uncertainty | 0.31** |  |  |  |  |
| Illness Perceptions  Timeline  Coherence  Personal Responsibility  Seriousness  Impact |  |  |  | 0.05 0.05 -0.02 -0.02 0.11* | 0.05 0.14* 0.01 -0.17* 0.13* |
| IPQ-R  Psychological Cause  Identity  Cyclical Timeline  Chronic Timeline  Consequences  Personal Control  Treatment control   Illness Coherence |  | 0.44* 0.14* 0.07 0.10 0.15 | -.09* 0.16** -.02 -.01 -.08* |  |  |
| Metacognitive Beliefs  PMC  NMC  CC  CSC  NC | 0.25** **0.45**** 0.27** 0.14 0.04 | 0.15* **0.44**** | 0.01 **0.54**** 0.03 0.06 0.06 | 0.09 **0.62**** 0.13* -0.02 0.08 | 0.02 **0.73**** 0.03 0.02 0.00 |

**Note:** IPQ-R = Illness Perceptions Questionnaire- Revised; NMC = Negative Metacognitive Beliefs (uncontrollability and danger of worry); CC = Cognitive Confidence; CSC = Cognitive Self Consciousness; PMC = Positive Metacognitive Beliefs; NC = Need for Control;** = p < 0.001; * = p < 0.05; bold = strongest predictor of anxiety symptoms
