# Supplementary material for: Metacognitive beliefs and their relationship with anxiety and depression in physical illnesses: A systematic review
Source: PLoS One. 2020 Sep 10;15(9):e0238457. doi: 10.1371/journal.pone.0238457 (PMC7500039; doi:10.1371/journal.pone.0238457)
Supplement: S5 Table — (DOCX) [file pone.0238457.s007.docx]

**S5**. Cognitive and Metacognitive Predictors of Depression

|  | Cook et al (2015) | Fisher, Reilly & Noble (2018) | Purewal & Fisher (2018) | |
| --- | --- | --- | --- | --- |
|  | Cancer (β) | Epilepsy (β) | Type 1 Diabetes (β) | Type 2 Diabetes (β) |
| Illness Perceptions  Timeline  Coherence  Personal Responsibility  Seriousness  Impact |  |  | 0.08 0.02 -0.13 0.06 0.15* | 0.07 0.17* -0.13 0.04 0.18* |
| IPQ-R  Psychological Cause  Identity  Cyclical Timeline  Chronic Timeline  Consequences  Personal Control  Treatment control   Illness Coherence | 0.14* 0.11 0.17* | -0.04 -0.08  0.18** 0.09 -0.14* 0.01 |  |  |
| Metacognitive Beliefs  PMC  NMC  CC  CSC  NC | 0.06 **0.29**** | -0.08 **0.23**** 0.19** -0.12* 0.23** | 0.05 **0.53**** 0.23** -0.03 0.23* | -0.19 **0.71**** 0.26* 0.14 0.05 |

**Note:** IPQ-R = Illness Perceptions Questionnaire- Revised; NMC = Negative Metacognitive Beliefs (uncontrollability and danger of worry); CC = Cognitive Confidence; CSC = Cognitive Self Consciousness; PMC = Positive Metacognitive Beliefs; NC = Need for Control;** = p < 0.001; * = p < 0.05; bold = strongest predictor of depression symptoms
